# Data from comprehensive analysis of nuclear localization signals

**DOI:** 10.1016/j.dib.2015.11.064

**Published:** 2015-12-12

**Authors:** Ryosuke Yamagishi, Hiroki Kaneko

**Affiliations:** a Department of Integrated Sciences in Physics and Biology, College of Humanities and Sciences, Nihon University, 3-25-40 Sakurajousui, Setagaya, Tokyo 156-8550, Japan; b National Institutes of Biomedical Innovation, Health and Nutrition, 7-6-8 Saito-Asagi, Ibaraki, Osaka 675-0085, Japan

**Keywords:** Nuclear localization signal, Nuclear transport, Comprehensive analysis, UniProt, Distribution

## Abstract

This article describes data related to a research article titled “Comprehensive analysis of the dynamic structure of nuclear localization signals” by Yamagishi et al. [1]. In this article, we provide the data covering wider range of the mammalian NLSs in UniProt (Universal Protein Resource) [2] regardless of their conformations. To be more specific as follows: We have extracted all NLSs which are clearly indicated as “NLS” with evidence type (a code from the Evidence Codes Ontology) [3] in UniProt. A total of 1364 NLSs in 1186 proteins were extracted from UniProt. The number of NLSs found in each protein (UniProt ID), the sequence length of NLSs and their distribution are shown.

Specifications Table

**Table:** 

Subject area	*Biology*
More specific subject area	*Molecular biology, Cell biology, Nuclear transport*
Type of data	*Table, graph*
How data was acquired	*Database analysis*
Data format	*Analyzed*
Experimental factors	*Analysis of information on mammalian nuclear localization signals identified in UniProt*
Experimental features	*We have extracted all NLSs which are clearly indicated as “NLS” with evidence type (a code from the Evidence Codes Ontology) in UniProt. We analyzed the number of NLSs found in each protein (UniProt ID) and their distribution and the sequence length of NLSs and their distribution.*
Data source location	
Data accessibility	*Data are accessible in this article*

## 1 Value of the data

• A total of 1364 NLSs in 1186 proteins were extracted from UniProt. The data have value in providing only accurate NLS information that is clearly indicated as “NLS” with evidence type (a code from the Evidence Codes Ontology) in UniProt. Therefore the data can be used as a training set for the development of NLS prediction programs.
• The data presented here are useful for researchers who study NLS and nuclear transport mechanism.
• Future studies concerning development of new therapeutic agents for human diseases caused by deregulation of nuclear transport such as numerous cancers and developmental disorders would require the data included here and the features of mammalian NLSs shown by the data.

## 2 Data

**Fig.1:**
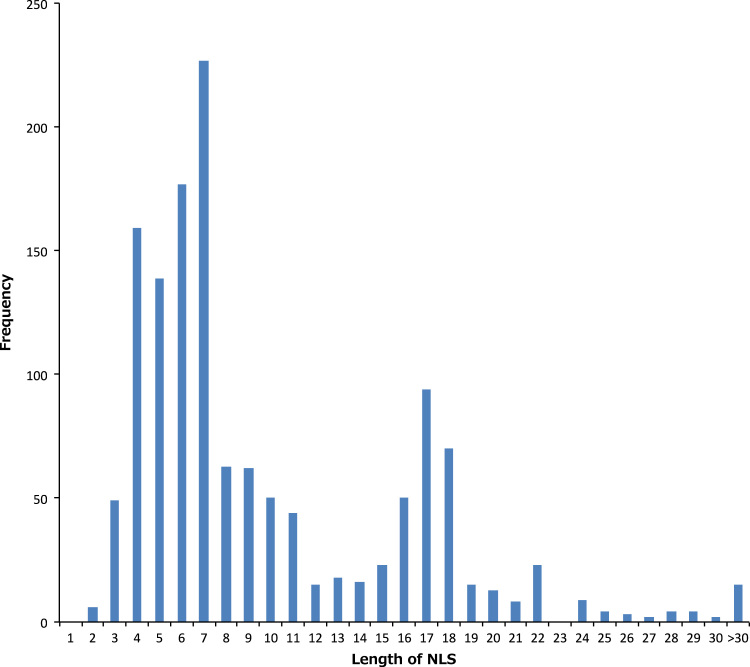
Histogram of sequence length of NLS.

**Fig. 2:**
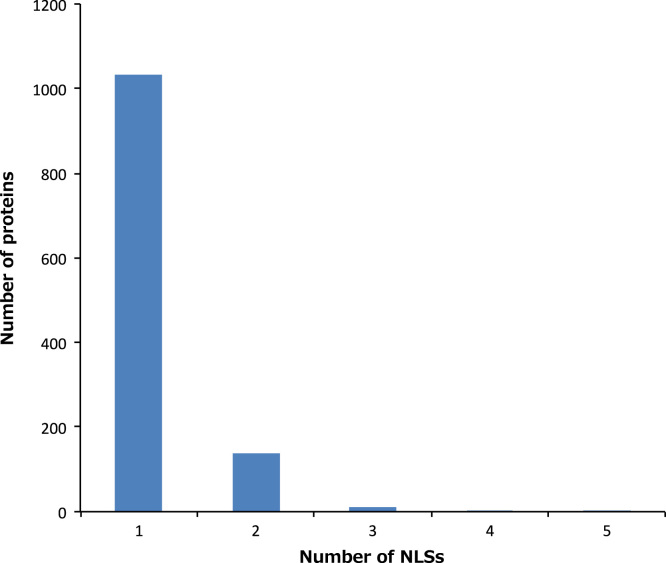
Histogram of the number of NLSs in one protein molecule.

In summary, a total of 1364 NLSs in 1186 proteins were extracted from UniProt. Data of individual NLSs are shown in Supplementary Table S1. The distribution of the length showed two peaks: one at 4–7 and one at 16–18, indicating the presence of monopartite and bipartite classical NLSs. The ratio of the NLSs consist of more than 30 residues was 1.10% (15/1364). We analyzed the sequence length of the NLSs and their distribution (Fig. 1). The numbers of NLSs found in one protein are also given in Supplementary Table S2. We analyzed the distribution of the number of NLSs in one protein molecule (Fig. 2). The proteins having only one NLS site were in large part and the ratio of the part was 86.93% (1031/1186). The numbers of proteins having two, three, four and five NLS sites were 138, 12, 4 and 1 in 1186, respectively.

## 3 Experimental design, materials and methods

### 3.1 Extraction of proteins with NLSs

In order to obtain the proteins having NLSs, UniProt (http://www.uniprot.org/) was used. We chose proteins　whose “Nuclear localization signal” is described in the “Description” of the item, and that are categorized in Mammalia. In more detail, we extracted the proteins from UniProt that satisfy the following conditions: annotation: (type:motif AND “nuclear localization signal”) AND taxonomy: “Mammalia [40674]” AND reviewed: yes. The UniProt IDs of the proteins having NLSs were obtained by this means.

### 3.2 Acquisition of positional information of NLSs

Positional information of NLSs, NLSs amino acid sequence information and Evidence Codes Ontology (ECO) were acquired from UniProt. In more detail, we obtained the information based on the following protocols:

1. Acquisition of XML formatted UniProt information from “http://www.uniprot.org/uniprot/[uniprot_id of protein with NLS].xml”.
2. Acquisition of protein name from the element with the tag <fullName> for each ID.
3. Search for the feature type tag as follows: Attribute type=“short sequence motif” AND Attribute description=“Nuclear localization signal”.
4. Acquisition of the start and end positions of NLS from the element with the tag<begin position> and <end position>.
5. Extraction of NLS amino acid sequence from the whole protein sequence based on its start and end positions.
6. Acquisition of ECO number from the element with the tag <evidence>.

The length of NLS was calculated from its start and end positions. Each evidence is described by a code from the Evidence Codes Ontology (ECO) as follows: ECO:0000250=By similarity, ECO:0000255=Sequence Analysis, ECO:0000269=Publication and ECO:0000305=Curated. The data on UniProt ID, protein name, start and end positions, sequence, length, evidence, ECO code for all NLSs were summarized in Supplementary Table S1. The histogram (Fig. 1) was produced based on the frequency distribution of the length of each NLS in Supplementary Table S1.

### 3.3 Count of the number of NLSs in one protein molecule

Some proteins have more than one NLS. Then we counted the number of NLSs in one polypeptide chain for each protein from the data shown in Supplementary Table S1. The data on UniProt ID, protein name and the number of NLSs were summarized in Supplementary Table S2. The histogram (Fig. 2) was produced based on the frequency distribution of the number of NLSs in each protein in Supplementary Table S2.

### 3.4 Programming

Python 2.6.6 was used as the programming language on Windows-based workstations.
